# Identification of anti-SARS-CoV-2 agents based on flavor/fragrance compositions that inhibit the interaction between the virus receptor binding domain and human angiotensin converting enzyme 2

**DOI:** 10.1371/journal.pone.0279182

**Published:** 2022-12-19

**Authors:** Yasumitsu Nishimura, Kenta Nomiyama, Shuichiro Okamoto, Mika Igarashi, Yusuke Yorifuji, Yukino Sato, Ayasa Kamezaki, Aya Morihara, Futoshi Kuribayashi, Akira Yamauchi

**Affiliations:** 1 Department of Hygiene, Kawasaki Medical School, Kurashiki, Okayama, Japan; 2 Shiono Koryo Kaisha, LTD, 1-6 Doshomachi 3-Chome, Chuo-ku, Osaka, Japan; 3 Department of Biochemistry, Kawasaki Medical School, Kurashiki, Okayama, Japan; Ehime University Graduate School of Medicine, JAPAN

## Abstract

Coronavirus disease 2019 (COVID-19) pandemic poses a threat to human beings and numerous cases of infection as well as millions of victims have been reported. The binding of the severe acute respiratory syndrome coronavirus 2 (SARS-CoV-2) spike protein receptor binding domain (RBD) to human angiotensin converting enzyme 2 (hACE2) is known to promote the engulfment of the virus by host cells. Employment of flavor/fragrance compositions to prevent SARS-CoV-2 infection by inhibiting the binding of viral RBD (vRBD) to hACE2 might serve as a favorable, simple, and easy method for inexpensively preventing COVID-19, as flavor/fragrance compositions are known to directly interact with the mucosa in the respiratory and digestive systems and have a long history of use and safety assessment. Herein we report the results of screening of flavor/fragrance compositions that inhibit the binding of vRBD to hACE2. We found that the inhibitory effect was observed with not only the conventional vRBD, but also variant vRBDs, such as L452R, E484K, and N501Y single-residue variants, and the K417N+E484K+N501Y triple-residue variant. Most of the examined flavor/fragrance compositions are not known to have anti-viral effects. Cinnamyl alcohol and Helional inhibited the binding of vRBD to VeroE6 cells, a monkey kidney cell line expressing ACE2. We termed the composition with inhibitory effect on vRBD-hACE2 binding as “the molecularly targeted flavor/fragrance compositions”. COVID-19 development could be prevented by using these compositions with reasonable administration methods such as inhalation, oral administration, and epidermal application.

## Introduction

Cases of novel corona virus infection (COVID-19) were first reported in Wuhan in December 2019 [1–3]; since then, millions of individuals have been affected worldwide. A virus named severe acute respiratory syndrome coronavirus 2 (SARS-CoV-2) has been reported as the cause of the pandemic and the mechanism by which SARS-CoV-2 infects to human cells has been investigated. The spike protein expressed on the envelope of SARS-CoV-2 plays a crucial role during the initial step of infection. The viral receptor binding domain (vRBD), which comprises amino acid residues 319–541 of the spike protein, is exposed to the outside environment; it binds to human angiotensin converting enzyme 2 (hACE2) expressed on the surface of host cells to promote the engulfment of the virus by the host cells [4–6]. Recently, a number of variants of SARS-CoV-2 have been reported, and some of them have been designated as variants of concern (VOCs) [7]. World Health Organization (WHO) also proposed labels for VOCs, such as alpha, beta, gamma and delta, whose vRBD contain N501Y, K417N/E484K/N501Y, K417T/E484K/N501Y, L452R/T478K, respectively (as presented in the European Centre for Disease Prevention and Control website; https://www.ecdc.europa.eu/en/covid-19/variants-concern). Studies have indicated that inhibiting the interaction of vRBD with hACE2 would be an effective strategy for preventing infection [5, 8, 9].

Flavors and fragrances have been used since ancient times and numerous functional flavors/fragrances are known to have favorable effects, such as relaxing and refreshing effects [10]. Most flavor/fragrance compositions are used as food additives, perfumes, and ingredients of daily commodities. As there is a long history of use of flavor/fragrance compositions and the associated safety assessments, most known flavor/fragrance compositions are relatively safe and inexpensive. Furthermore, owing to the daily usage, most of the flavor/fragrance compositions have high possibilities of interacting with the mucosa of the respiratory and digestive systems. The use of flavor/fragrance compositions for people with a high risk of SARS-CoV-2 infection would be a simple and inexpensive approach to prevent COVID-19 development without causing any major physiological alternations.

In the present study, we screened flavor/fragrance compositions and identified some agents that inhibit binding of vRBD to hACE2 and binding of vRBD to monkey kidney VeroE6 cells expressing ACE2.

## Materials and methods

### Reagents and instruments

All reagents were purchased from Sigma-Aldrich Co. LLC. (St. Louis, MO, USA), a part of Merck KGaA (Darmstadt, Germany), unless otherwise mentioned. Clear-flat bottom 96 well plates were from Corning Inc. (Corning, NY, USA). VeroE6/TMPRSS2 cells, an ACE2 expressing monkey kidney cell line, which was transduced to stably express serine protease TMPRSS2, were procured from JCRB cell bank (JCRB1819; Ibaraki, Osaka, Japan) [11, 12].

### Flavor and fragrance compositions

Flavor and fragrance essences and chemicals were obtained from commercially available compound libraries. A total of 331 different kinds of agents (59 natural extracts and 272 compounds) (S1 and S2 Tables, respectively) were tested in this study as representative flavor/fragrance compositions.

### Inhibitor screening

The inhibitory effect of the flavor/fragrance compositions was evaluated using the SARS-CoV-2 Inhibitor screening Kit (AcroBIOSYSTEMS, Newark, DE, USA), which contains conventional (Wuhan type) SARS-CoV-2 RBD and wild-type human ACE2, according to the manufacturer’s protocol with slight modifications. Briefly, the recombinant protein (vRBD) was coated on clear-flat bottom 96-well plates; then, the plates were blocked with 2% (w/v) bovine serum albumin (BSA) prepared in washing buffer (PBS containing 0.05% (v/v) Tween-20, pH 7.4). Subsequently, flavor/fragrance compositions and biotinylated ACE2 prepared in buffer (1.5% (v/v) Tween-20, pH 7.4 and 0.5% (w/v) BSA) were added to the plates, which were then incubated for 1h at 37°C. Thereafter, the flavor/fragrance compositions and hACE2 were washed out vigorously using the wash buffer. After incubation with streptavidin-tagged horseradish peroxidase (prepared in dilution buffer [0.05% (v/v) Tween-20, pH 7.4 and 0.5% (w/v) BSA]), TMB substrate solution (20 mg/mL 3,3′,5,5′-Tetramethylbenzidine (TMB) in 50 mM disodium hydrogen phosphate and 25 mM citric acid, pH 5.5), was added to visualize the binding of vRBD and hACE2, and then the reaction was stopped by adding 1 M sulfuric acid. The absorbance of the supernatant in each well was measured using the Varioskan microplate reader (Thermo Fisher Scientific, Waltham, MA, USA) at a wavelength of 450nm. In some experiments, the recombinant conventional vRBD protein was replaced with histidine-tagged recombinant variant vRBD proteins containing an L452R, E484K, or N501Y single-residue variation, a K417N+E484K+N501Y triple-residue variation (3mut), or an Omicron variant (BA.1.1.529) (residue 319–537, 26.6kDa, each) (AcroBIOSYSTEMS, Newark, DE, USA).

The statistical analysis was performed using EZR software (ver.1.5), a modified version of R commander designed to add statistical functions frequently used in biostatistics [13]: Mean values ± SD are shown in graphs, and the analysis was performed using Student’s *t*-test.

### Flow cytometry

The inhibitory effect of the flavor/fragrance compositions at the cellular level was evaluated using recombinant histamine-tagged vRBD protein and VeroE6/TMPRSS2 cells. Briefly, 1 × 10^6^ VeroE6/TMPRSS2 cells were incubated with 2 μg/mL vRBD in 100-μL volume for 30 min with or without different concentrations of the flavor/fragrance compositions. Thereafter, the cells were washed with 1 mL of phosphate buffered saline containing 0.1% bovine serum albumin (0.1% BSA/PBS) at 250 × g for 5 min at 4°C, followed by incubation with murine anti-Histidine tag antibody at 1 μg/mL in 100-μL volume for 30 min. The cells were the washed with 1 mL of 0.1% BSA/PBS at 250 × g for 5 min at 4°C, and then incubated with anti-mouse immunoglobulin antibody conjugated with Alexa488 (MBL, Tokyo, Japan). After washing with PBS at 250 × g for 5 min at 4°C, the cells were resuspended in 500 μL of 0.1% BSA/PBS and analyzed using the FACS Canto II flow-cytometer (Becton Dickinson, NJ, USA). Data were analyzed using FACS DIVA software ver. 8.0.2 (Becton Dickinson, NJ, USA).

## Results

### Screening of flavor/fragrance compositions that inhibit the binding of vRBD to hACE2

**Fig 1:**
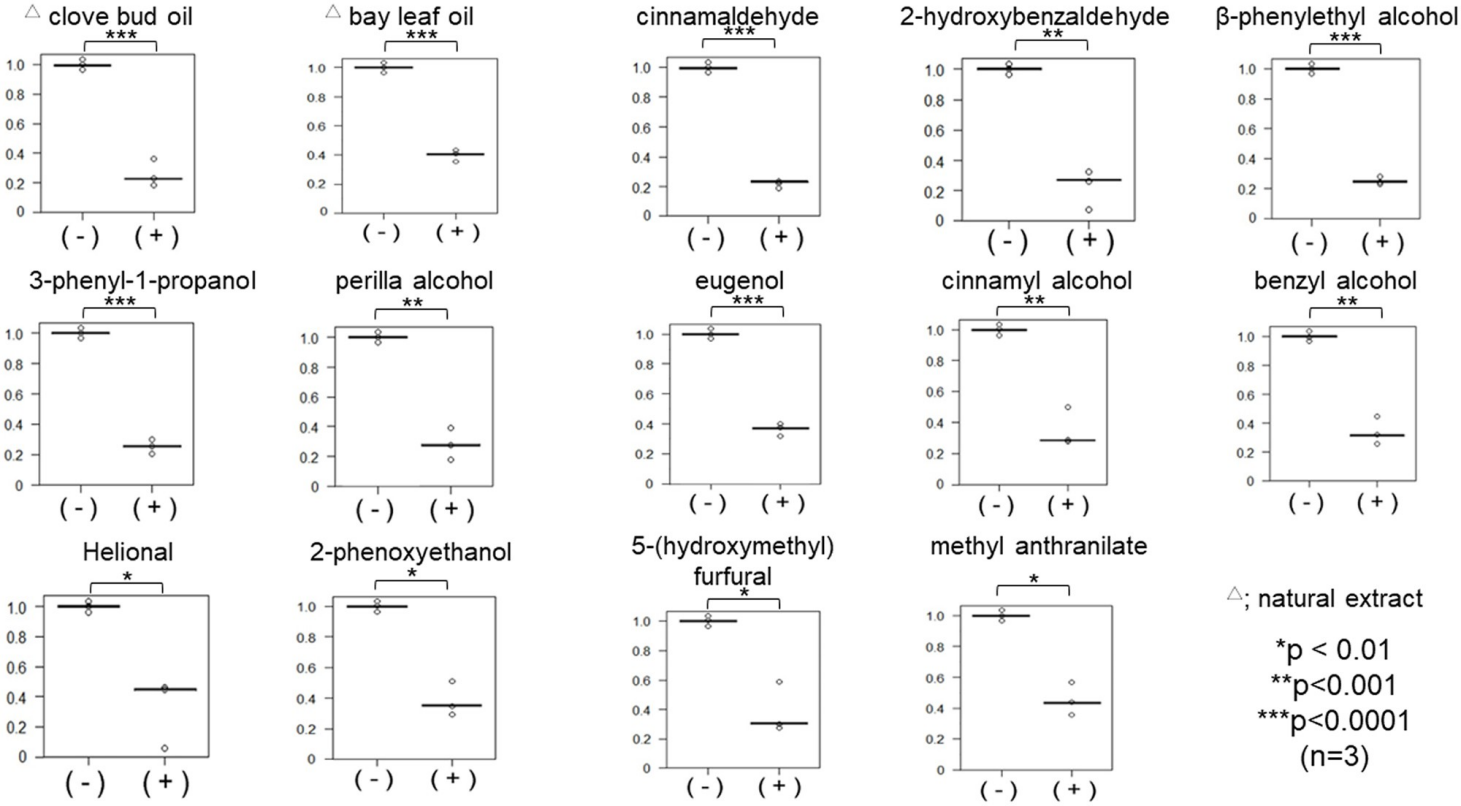
Inhibition of the binding of SARS-CoV-2 RBD to hACE2 by flavor/fragrance compositions. The relative binding of recombinant SARS-CoV-2 RBD to recombinant hACE2 was evaluated in 96 well plates with or without 0.5% (v/v) flavor/fragrance compositions; 0.5% DMSO was used as the solvent for each flavor/fragrance composition and 0.5% DMSO served as the negative control (= 1.0). Three independent experiments were performed. Statistical differences between DMSO control (-) and DMSO with flavor/fragrance composition (+) were evaluated using Student’s *t*-test (n = 3 *p<0.01, **p<0.001, ***p<0.0001). Triangles(△) indicate natural extracts.

To screen effective flavor/fragrance compositions from natural extracts and flavor/fragrance chemicals as inhibitors of binding between vRBD and hACE2, we investigated more than 300 kinds of existing flavor/fragrance compositions (59 natural extracts and 272 compounds) (S1 and S2 Tables, respectively) at a concentration of 0.5% (v/v) in the first screening. We found that 4 kinds of extracts and 22 compounds had more than 50% inhibitory effect on vRBD-hACE2 binding (Table 1). We confirmed that 2 kinds of extracts and 12 kinds of single-agents have significant inhibitory effect on the binding between vRBD and hACE2 (Fig 1). We used the concentration of 0.5% (v/v) because most of the scented products contain 0.1%–2% fragrance composition. Indeed, some products contain fragrance compositions up to 20% [14].

**Table 1:** Ranking of flavor/fragrance compositions in the first screening.

ranking at 1^st^ screening	agents name	CAS	relative vRBD-hACE2 binding
1	2-hydroxybenzaldehyde (= salicylaldehyde)	90-02-8	0.077
2	Cinnamaldehyde	104-55-2	0.239
3	β-Phenylethyl alcohol	60-12-8	0.283
4	3-phenyl-1-propanol	122-97-4	0.298
5	5-(hydroxymethyl)furfural	67-47-0	0.305
6	Oakmoss abs.*	520-43-4	0.306
		4707-47-5	
7	Methyl anthranilate	134-20-3	0.355
8	Clove bud oil *	97-53-0	0.364
		93-28-7	
		87-44-5	
9	Shogaol	555-66-8	0.378
10	perilla alcohol (= p-1,8-menthadien-7-ol)	536-59-4	0.391
11	Eugenol	97-53-0	0.397
12	dihydroactinidiolide	15356-74-8	0.399
13	Ald. C18 γ-Nonalactone	104-61-0	0.406
14	Caraway oil *	2244-16-8	0.424
		5989-27-5	
		99-48-9	
15	Bay leaf oil *	97-53-0	0.427
		127-91-3	
		93-15-2	
16	Ald. C11(Lenic)	112-45-8	0.448
17	benzyl alcohol	100-51-6	0.449
18	€-4-decenal	65405-70-1	0.460
19	Helional (2-Methyl-3-(3,4-Methylenedioxyphenyl)Propanal)	1205-17-0	0.461
20	p-1-menthen-9-ol	18479-68-0	0.462
21	α-fenchol (= α-fenchyl alcohol)	14575-74-7	0.468
22	Methyl atrarate	4707-47-5	0.478
23	Citral	5392-40-5	0.482
24	Aurantin	522-16-7	0.487
25	Methyl β-naphthyl ketone	93-08-3	0.494
26	Cinnamyl alcohol	104-54-1	0.496
27	2-phenoxyethanol	122-99-6	0.507
28	Anisaldehyde	123-11-5	0.519
29	Methyl isoeugenol	93-16-3	0.523
30	Terpineol	98-55-5	0.526
31	Lyral	31906-04-4	0.527
32	4-terpineolerpinenenen-4-ol)	562-74-3	0.543
33	Benzyl acetate	140-11-4	0.549
34	Indole	120-72-9	0.556
35	3,6-nonadien-1-ol	76649-25-7	0.564
36	Raspberry ketone	5471-51-2	0.568
37	Ald. C12(Lauric)	112-54-9	0.571
38	Ylang oil *	78-70-6	0.573
		104-93-8	
		140-11-4	
		93-58-3	
39	Ald. C12 MNA	110-41-8	0.574
40	(Z)-6-nonen-1-ol	35854-86-5	0.577
41	gingerone	122-48-5	0.593
42	Orange oil sweet *	78-70-6	0.597
		7212-44-4	
		115-95-7	
		120-72-9	
		134-20-3	
43	Vetiver oil *	89-88-3	0.607
		15764-04-2	
44	β-Phenylethyl acetate	103-45-7	0.614
45	Coumarin	91-64-5	0.622
46	(Z)-6-nonenal	2277-19-2	0.625
47	Violet leaf abs. *	28069-72-9	0.625
		557-48-2	
48	2-methoxy-3-methylpyrazine	2847-30-5	0.630
49	Isoamyl acetate	123-92-2	0.640
50	β-Damascone	35044-68-9	0.645
51	Lily aldehyde	80-54-6	0.653
52	cis-3-Hexenol	928-96-1	0.661
53	Benzaldehyde glyceryl acetal	1319-88-6	0.668
54	Basil oil *	140-67-0	0.677
		78-70-6	
		93-15-2	
55	Ethyl 3-phenylglycidate	121-39-1	0.679
56	Ethyl butyrate	105-54-4	0.686
57	1-nonanol	143-08-8	0.691
58	Methyl benzoate	93-58-3	0.695
59	(Z)-4-decen-1-ol	57074-37-0	0.697
60	Celery seed oil *	5989-27-5	0.700
		17066-67-0	
		6066-49-5	
61	Maltol	118-71-8	0.702
62	Patchouli oil *	5986-55-0	0.710
		560-32-7	
		88-84-6	
63	Orange flower abs. *	5989-27-5	0.712
		112-31-2	
		4630-07-3	
64	1,10-dihydronootkatone	20489-53-6	0.715
65	Allyl amylglycolate	67634-00-8	0.718
66	Lavender oil *	78-70-6	0.736
		115-95-7	
		25905-14-0	
67	Heliotropin	120-57-0	0.736
68	piperonyl acetate	326-61-4	0.747
69	Ald. C10	112-31-2	0.751
70	9-decen-1-ol	13019-22-2	0.755
71	α-Ionone	127-41-3	0.757
72	4-hydroxybenzaldehyde	123-08-0	0.761
73	Nootkatone	4674-50-4	0.762
74	Borneol	507-70-0	0.763
75	Ald. C16 Ethyl methylphenylglycidate	77-83-8	0.763
76	Ethyl vanillin	121-32-4	0.765
77	Elemi res. *	639-99-6	0.766
		487-11-6	
78	Camphor	464-49-3	0.771
79	Galbanum res. *	489-86-1	0.774
		51411-24-6	
80	Citronellol	106-22-9	0.780
81	Vanillin	121-33-5	0.789
82	Methyl cinnamate	103-26-4	0.793
83	Pine needle oil *	80-56-8	0.793
		79-92-5	
		76-49-3	
84	Myrrh res. *	20307-84-0	0.795
		3856-25-5	
85	Damascenone	23696-85-7	0.798
86	Cinnamon bark oil *	104-55-2	0.799
		97-53-0	
87	α-Hexylcinnamaldehyde	101-86-0	0.799
88	cis-Jasmone	488-10-8	0.805
89	Armoise oil *	546-80-5	0.807
		464-49-3	
90	3-carene	13466-78-9	0.808
91	Coriander oil *	78-70-6	0.809
		464-49-3	
		507-70-0	
92	Neroli oil *	78-70-6	0.809
		115-95-7	
		7212-44-4	
93	α-Isomethyl ionone	127-51-5	0.821
94	Orris concrete	79-68-5	0.821
		79-69-6	
		544-63-8	
95	Ambroxan	6790-58-5	0.822
96	Civet abs. *	542-46-1	0.822
97	sec-Butyl quinoline	67634-06-4	0.827
98	Galbanum oil *	127-91-3	0.833
		19883-29-5	
99	ESTRAGOLE	140-67-0	0.838
100	1-decanol	112-30-1	0.839

* natural products

In this study, 59 natural extracts and 272 compounds (total 331) listed in S1 and S2 Tables were examined in the first screening. The top 100 effective flavor/fragrance compositions are listed here. Relative vRBD-hACE2 binding indicates the binding activity between vRBD and hACE2 under different compositions compared with that under 0.5% DMSO control. (1.000 indicates 100% binding and 0.500 indicates 50% binding).

### Flavor/fragrance compositions effective against the binding between conventional vRBD and hACE2 prevent the interaction between mutant vRBDs and hACE2

**Fig 2:**
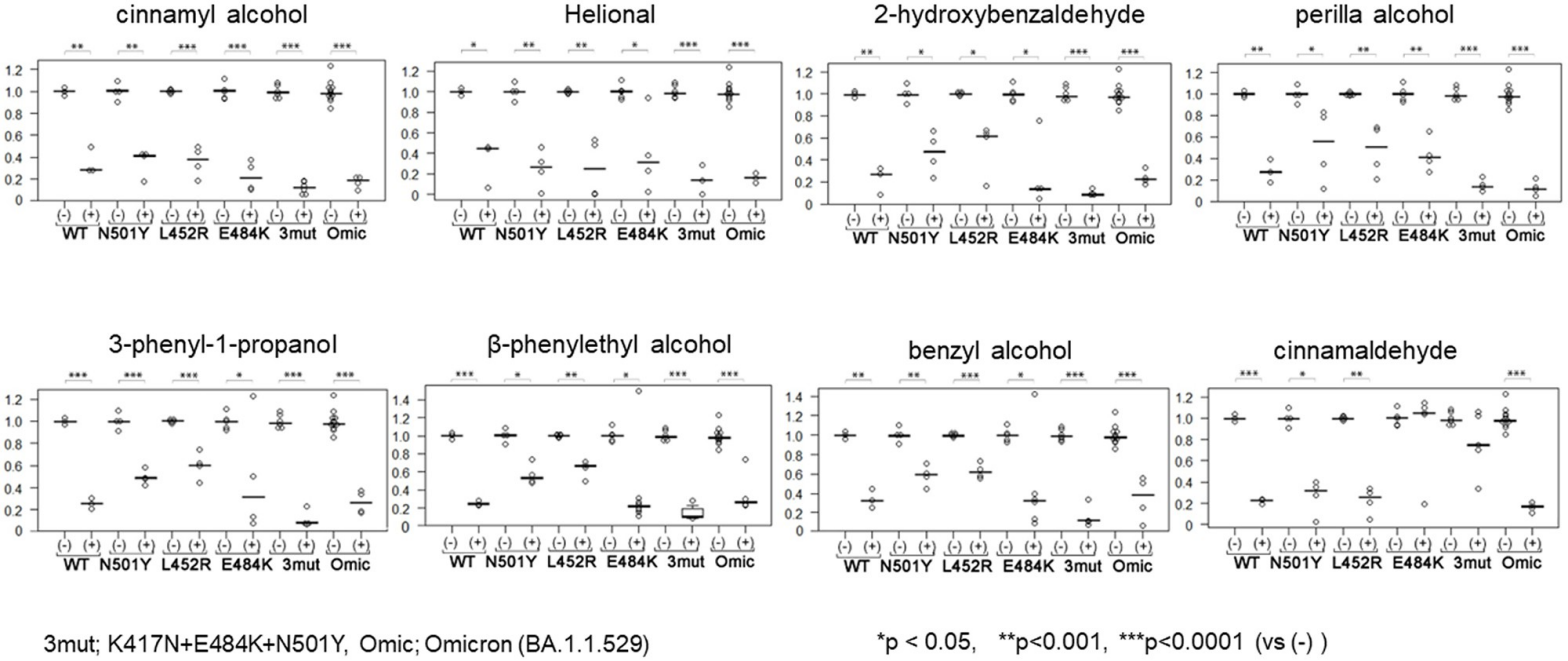
Inhibition of the binding of the variant vRBD to hACE2 by flavor/fragrance compositions. Relative binding of recombinant SARS-CoV-2 RBDs to recombinant hACE2 evaluated in 96-well plates with or without 0.5% (v/v) flavor/fragrance compositions: 0.5% DMSO was the solvent for each flavor/fragrance composition and used as the negative control (= 1.0). Three or more independent experiments were performed. Statistical differences between DMSO control and flavor/fragrance compositions prepared in DMSO compared using Student’s *t*-test. WT; wild-type (= Wuhan type), 3mut; K417N+E484K+N501Y (n = 3 [WT], n≥3 [N501Y, L452R, E484K, 3mut, and Omicron], *p < 0.01, **p < 0.001, ***p < 0.0001 compared with 0.5% DMSO control).

During the COVID-19 pandemic, several SARS-CoV-2 variants have emerged and some of them have mutations in the vRBD. Therefore, 8 representative flavor/fragrance compositions were selected and evaluated for the inhibitory activity against the interaction between vRBD mutants and hACE2. We found that flavor/fragrance compositions effective against conventional (Wuhan type) vRBD-hACE2 interaction also exhibited inhibitory activity against the binding of variant vRBDs–the L452R, E484K, and N501Y single-residue variants, and the K417N+E484K+N501Y triple-residue variant (3mut) as well as the Omicron (BA.1.1.529) variant (Fig 2). In addition, some flavor/fragrance compositions were more effective against a certain kind of variant vRBD. For example, cinnamyl alcohol and Helional (Synonym: 2-Methyl-3-(3,4-Methylenedioxyphenyl)Propanal) effectively inhibited all kinds of vRBDs, whereas cinnamaldehyde inhibited the binding of conventional, N501Y, L452R and Omicron, but not that of E484K or 3 mut vRBD.

### Dose dependency of inhibition with respect to vRBD-hACE2 binding

**Fig 3:**
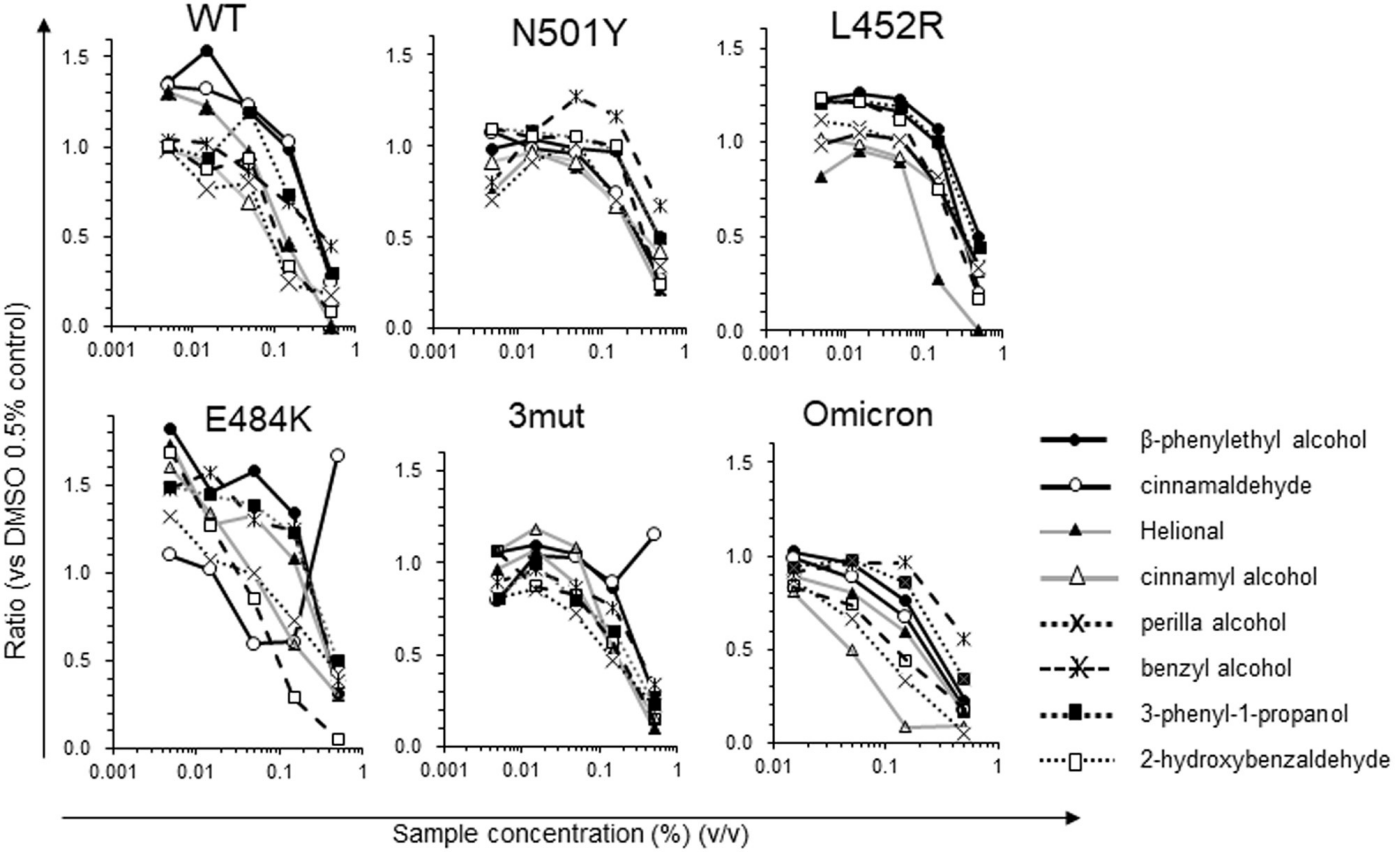
Dose dependency of flavor/fragrance compositions for the inhibition of the binding of variant vRBDs to hACE2. Five concentrations (0.5%, 0.15%, 0.05%, 0.015%, and 0.005%) of each flavor/fragrance composition were evaluated in 96-well plates. 0.5% DMSO was the solvent for each flavor/fragrance composition and was used as the negative control (= 1.0). Data presented are representative of two or more experiments.

The dose dependency of the inhibitory effect of the flavor/fragrance compositions was also examined using sequentially diluted samples of conventional and variant vRBDs. The eight types of agents showed an inhibitory effect against each vRBD-hACE2 binding in a dose-dependent manner, at least with three doses, except cinnamaldehyde for E484K single-residue variant and K417N+E484K+N501Y triple-residue variant (Fig 3).

### Comparing total inhibitory activity among the anti-COVID-19 flavor/fragrance compositions

**Fig 4:**
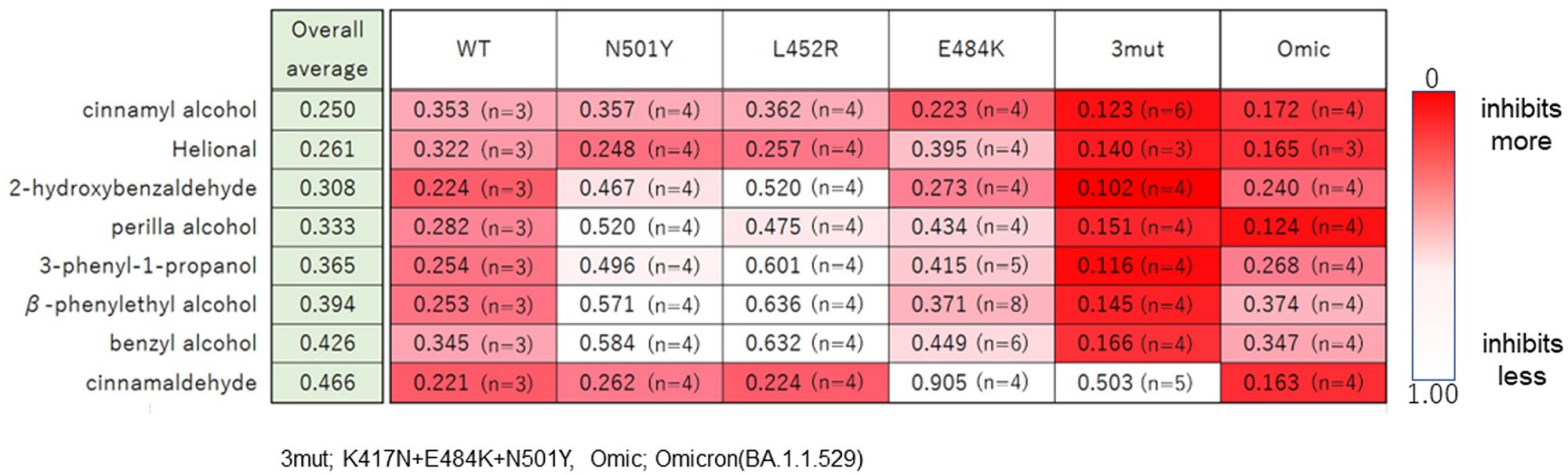
Ranking and heatmap of the top eight flavor/fragrance compositions inhibiting the binding between hACE2 and vRBDs. The averages were calculated from each value corresponding to vRBD-hACE2 binding measured using vRBDs. Overall average column shows values of all data using WT and variants. Values in the heatmap are the vRBD-hACE2 binding activities measured using 0.5% (v/v) flavor/fragrance compositions. Samples with vigorous inhibition are in red.

To investigate the total inhibitory effects of the screened anti-COVID-19 flavor/fragrance compositions, we prepared a heatmap of the inhibitory activity (Fig 4). The inhibitory activity ranking is based on the overall average binding activity of all types of variants. Of these flavor/fragrance compositions, cinnamyl alcohol was the most effective against the binding of these conventional and vRBD to hACE2.

### Inhibitory activity of the anti-COVID-19 flavor/fragrance compositions with vRBD proteins and cells

**Fig 5:**
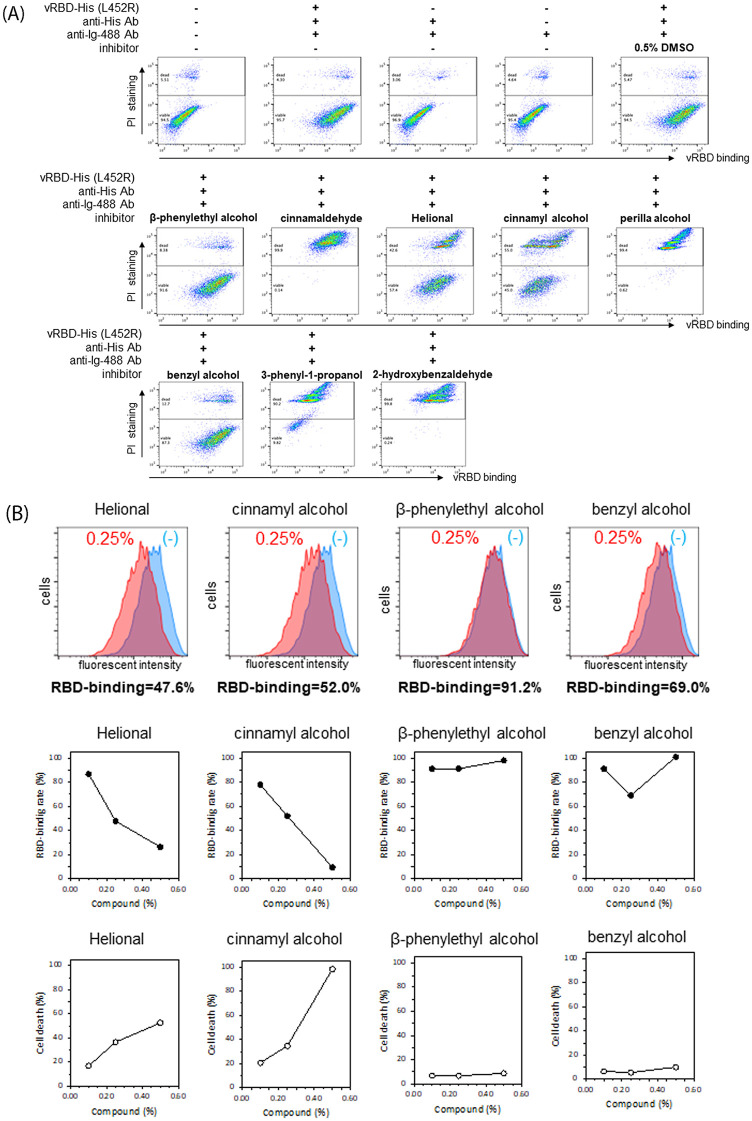
Analysis of inhibitory activities of flavor/fragrance compositions at the cellular level. Flow-cytometry was performed with histidine-tagged Omicron (BA.1.1.529) vRBD and the ACE2-expressing VeroE6/TMPRSS2 cells. (A) Dot plots of vRBD-bound VeroE6/TMPRSS2 cells. Cells were stained with propidium iodide (PI) to check viability as well as anti-histidine antibody, followed by detection with anti-immunoglobulin-Alexa488 fluorescence. PI-positive dead cells were plotted in the upper part of the plots. (B) [Top row] Histograms of vRBD-bound VeroE6/TMPRSS2 cells with (red) or without (light blue) flavor/fragrance compositions. RBD-binding rates are shown under the histogram. [Middle row] Binding rate at different concentrations of flavor/fragrance compositions. Relative binding values were calculated based on the negative control without flavor/fragrance compositions. [Bottom row] Viability of VeroE6/TMPRSS2 cells at various concentrations of flavor/fragrance compositions. Dead cells were determined using PI-staining. Data are representative of three independent experiments.

Not only protein–protein interactions but also protein–cell interactions were evaluated using recombinant histidine-tagged Omicron (BA.1.1.529) vRBD and the ACE2-expressing monkey kidney cell line VeroE6/TMPRSS2 (Fig 5A and 5B). Among the eight flavor/fragrance compositions selected by screening with the protein–protein interaction experiments, 4 of them (helional, cinnamyl alcohol, β-phenylethyl alcohol, and benzyl alcohol) were less cytotoxic and the other 4 (cinnamaldehyde, perilla alcohol, 3-phenyl-1-propanol and 2-hydroxybenzaldehyde) showed cytotoxicity (Fig 5A). Among the low cytotoxic compositions, helional and cinnamyl alcohol inhibited the binding of vRBD to VeroE6 cells in a dose-dependent manner. It is noteworthy that helional showed relatively low cytotoxicity and most potent inhibitory activity.

## Discussion

SARS-CoV-2 infects human cells via hACE2 which is expressed on epithelial cells of from the oral cavity to colon, kidney cells, cholangiocytes, Sertoli cells, and type II alveolar epithelial cells [15, 16]. The respiratory system and digestive system are the front line of viral defense; most of the flavor/fragrance compositions are thought to be internalized and absorbed via the respiratory and/or digestive systems because these compositions are used as ingredients of food or daily commodities. Therefore, the use of anti-coronaviral compositions presented here is a reasonable strategy for humans as the defensive rationale is to prevent the interaction between vRBD and hACE2 which is the first step in coronaviral infection.

We found 14 kinds of flavor/fragrance compositions that potentially inhibited vRBD-hACE2 binding (Fig 1) and at least 8 of them showed inhibitory activities on WT and variant vRBDs (Fig 2). Among these flavor/fragrance compositions, Helional and cinnamyl alcohol had the inhibitory activities on vRBD-ACE2 binding in protein–protein and protein–cell interactions (Figs 2 and 5). These flavor/fragrance compositions are good candidates for the prevention of COVID-19. Although some of the flavor/fragrance compositions selected by protein–protein interaction experiments showed toxicity against suspended bare cells in this study, these compositions have been are proven to be not-toxic *in vivo* and are still safe to use in daily life [17–20].

There are some limitations to this study. The results shown here indicate protein–protein and protein–cell interactions *in vitro*, thereby providing basic information for further investigations. To prove the usefulness of the flavor/fragrance compositions shown here and to discuss the prevention of viral infection, more evidence with respect to *in vivo* scenario, in addition to using the active virus, is required. Furthermore, vRBD examined here are included in WT and variants, such as alpha, beta, gamma, delta, and omicron(BA.1.1.529) strains but they do not cover other variants. The effect on the binding of recently emerged omicron type vRBD such as BA.5 with hACE2 is currently under investigation.

Importantly, the flavor/fragrance compositions examined here have been used in the real-world. Indeed, cinnamaldehyde and cinnamyl alcohol are abundantly present in *Cinnamomum spp*. such as *C*. *zeylanicum* and *C*. *cassia*, have been reported to have antimicrobial and antipyretic activities [21, 22]. Additionally, 2-hydroxybenzaldehyde is reported to be an ingredient of natural foods such as buckwheat and kiwifruit [23, 24]. Helional is known to be a specific ligand for the human odorant receptor OR 17–40 [25]. Here, we showed that flavor/fragrance compositions including cinnamyl alcohol and Helional have an inhibitory effect on the binding of SARS-CoV-2 RBD to hACE2, it can be called as “the molecularly targeted flavor/fragrance”.

Moreover, several compounds found in natural plants and food, such as tannin [26] and tannic acid [27, 28], have been reported to have anti-SARS-CoV-2 effects *in vivo* and *in vitro*, respectively. Therefore, food-derived compounds with anti-SARS-CoV-2 effects, including those found in our current study, are expected to be useful for prevention and treatment of COVID-19.

The findings would enable us to conduct epidemiological studies in the next phase. Moreover, flavors might have factors that could help to regulate viral infection, thereby limiting the prevalence or mortality rate in various countries and regions.

The WHO and the Health Service Bureau of individual countries have accepted vaccination as the master strategy to prevent COVID-19 spread (https://www.who.int/emergencies/diseases/novel-coronavirus-2019/covid-19-vaccines). However, some people cannot undergo vaccination because of medical issues such as allergic reactions and adverse reactions. It is always favorable to enhance the range of choices available to prevent the infection. The anti-coronaviral flavor/fragrance compositions shown here contribute to such choices. Furthermore, flavors and fragrances have advantages in terms of safety. The safety of most of the compositions has been established, as flavors and fragrances have a long history of use and the flavor/fragrance compositions shown here are well-known and well-used. Moreover, most of the flavor/fragrance compositions are available at a relatively low cost, compared with vaccines and biological medicines. Therefore, the strategy of using flavor/fragrance compositions to prevent COVID-19 spread has temporal and economic benefits.

In conclusion, we found several flavor/fragrance compositions that inhibit vRBD-hACE2 binding by screening numerous flavor/fragrance compositions *in vitro*, such as cinnamyl alcohol, Helional, 2-hydroxybenzaldehyde, perilla alcohol, 3-phenyl-1-propanol, β-Phenylethyl alcohol, benzyl alcohol, and Cinnamaldehyde. Of these compounds, cinnamyl alcohol and Helional would be the best candidates for an anti-COVID-19 agent because these two were shown to inhibit vRBD binding to VeroE6 cells.

## Supporting information

S1 Table Fifty-nine natural extracts evaluated in this study. The extract name, CAS number, and major component are shown.
(DOCX)

S2 Table Two hundred and seventy-two synthetic flavor/fragrances evaluated in this study. The flavor/fragrance name and CAS number are shown.
(DOCX)

## Abbreviations

BSA: bovine serum albumin
DMSO: dimethyl sulfoxide
hACE2: human angiotensin converting enzyme 2
Helional: 2-Methyl-3-(3,4-Methylenedioxyphenyl)Propanal
PBS: phosphate buffered saline
TMB: 3,3′,5,5′-tetramethylbenzidine
VOC: variant of concern
vRBD: viral receptor binding domain
WHO: World Health Organization
