# Supplementary material for: Identification of anti-SARS-CoV-2 agents based on flavor/fragrance compositions that inhibit the interaction between the virus receptor binding domain and human angiotensin converting enzyme 2
Source: PLoS One. 2022 Dec 19;17(12):e0279182. doi: 10.1371/journal.pone.0279182 (PMC9762593; doi:10.1371/journal.pone.0279182)
Supplement: S1 Table — The extract name, CAS number, and major component are shown. (DOCX) [file pone.0279182.s001.docx]

**S1 Table**

Natural extracts.

| No. | Sample name | CAS number | major component |
| --- | --- | --- | --- |
| 1 | Lemon oil | 5989-27-5 5392-40-5 124-19-6 | d-limonene citral nonanal |
| 2 | Bergamot oil | 5989-27-5 115-95-7 78-70-6 | d-limonene linalyl acetate linalool |
| 3 | Orange oil sweet | 78-70-6 7212-44-4 115-95-7 120-72-9 134-20-3 | linalool nerolidol linalyl acetate indole methyl anthranilate |
| 4 | Mandarin oil | 5989-27-5 85-91-6 | d-limonene methyl N-methylanthranilate |
| 5 | Lime oil distilled | 5989-27-5 98-55-5 99-85-4 | d-limonene α-terpineol γ-terpinene |
| 6 | Grapefruit oil | 5989-27-5 124-13-0 4674-50-4 | d-limonene octanal nootkatone |
| 7 | Sandalwood oil | 115-71-9 512-61-8 | santalol santalene |
| 8 | Cedarwood oil | 469-61-4 77-53-2 470-40-6 | α-cedrene cedrol thujopsene |
| 9 | Vetiver oil | 89-88-3 15764-04-2 | vetiverol vetivone |
| 10 | Patchouli oil | 5986-55-0 560-32-7 88-84-6 | patchouli alcohol patchoulene guaiene |
| 11 | Oakmoss abs. | 520-43-4 4707-47-5 | methyl everninate methyl β-orcinol carboxylate |
| 12 | Clove bud oil | 97-53-0 93-28-7 87-44-5 | eugenol acetoeugenol β-caryophyllene |
| 13 | Cinnamon bark oil | 104-55-2 97-53-0 | cinnamaldehyde eugenol |
| 14 | Bay leaf oil | 97-53-0 127-91-3 93-15-2 | eugenol β-pinene methyl eugenol |
| 15 | Nutmeg oil | 80-56-8 3387-41-5 607-91-0 | α-pinene sabinene myristicin |
| 16 | Pepper oil | 80-56-8 138-86-3 87-44-5 | pinene limonene β-caryophyllene |
| 17 | Neroli oil | 78-70-6 115-95-7 7212-44-4 | linalool linalyl acetate nerolidol |
| 18 | Petitgrain oil | 78-70-6 115-95-7 105-87-3 | linalool linalyl acetate geranyl acetate |
| 19 | Orange flower abs. | 5989-27-5 112-31-2 4630-07-3 | d-limonene decanal valencene |
| 20 | Fennel sweet oil | 4180-23-8 123-11-5 1195-79-5 | anethole anis aldehyde fenchone |
| 21 | Basil oil | 140-67-0 78-70-6 93-15-2 | estragole linalool methyl eugenol |
| 22 | Caraway oil | 2244-16-8 5989-27-5 99-48-9 | d-carvone d-limonene carveol |
| 23 | Rose abs. | 7540-51-4 106-24-1 16409-43-1 35044-68-9 | l-citronellol geraniol rose oxide β-damascone |
| 24 | Rose oil | 7540-51-4 106-24-1 60-12-8 | l-citronellol geraniol β-phenylethyl alcohol |
| 25 | Geranium oil | 7540-51-4 106-24-1 491-07-6 | l-citronellol geraniol isomenthone |
| 26 | Jasmin abs. | 140-11-4 120-72-9 488-10-8 | benzyl acetate indole cis-jasmone |
| 27 | Narcissus abs. | 13877-91-3 93-16-3 | ocimene methyl isoeugenol |
| 28 | Ylang ylang oil | 78-70-6 104-93-8 140-11-4 93-58-3 | linalool methyl p-cresol benzyl acetate methyl benzoate |
| 29 | Orris concrete | 79-68-5 79-69-6 544-63-8 | γ-irone α-irone myristic acid |
| 30 | Lavender oil | 78-70-6 115-95-7 25905-14-0 | linalool linalyl acetate lavandulyl acetate |
| 31 | Rosemary oil | 470-82-6 507-70-0 464-49-3 | 1,8-cineole borneol camphor |
| 32 | Thyme oil | 89-83-8 499-75-2 99-87-6 | thymol carvacrol p-cymene |
| 33 | Eucalyptus oil | 470-82-6 | 1,8-cineole |
| 34 | Laurel leaf oil | 470-82-6 562-74-3 93-15-2 | 1,8-cineole terpinen-4-ol methyl eugenol |
| 35 | Peru balsam oil | 120-51-4 103-41-3 7212-44-4 121-33-5 | benzyl benzoate benzyl cinnamate nerolidol vanillin |
| 36 | Tolu balsam res. | 120-51-4 103-36-6 | benzyl benzoate ethyl cinnamate |
| 37 | Vanilla extract. | 121-33-5 | vanillin |
| 38 | Styrax res. | 122-69-0 122-68-9 104-54-1 | cinnamyl cinnamate phenylpropyl cinnamate cinnamyl alcohol |
| 39 | Labdanum res. | 2021-28-5 | ethyl dihydrocinnamate |
| 40 | Clary sage oil | 115-95-7 78-70-6 515-03-7 | linalyl acetate linalool sclareol |
| 41 | Olibanum res. | 60305-17-1 99-87-6 80-57-9 | δ-cadinene p-cymene verbenone |
| 42 | Benzoin res. | 65-85-0 121-33-5 93-89-0 | benzoic acid vanillin ethyl benzoate |
| 43 | Opoponax res. | 17627-44-0 | α-bisabolene |
| 44 | Myrrh res. | 20307-84-0 3856-25-5 | δ-elemene α-copaene |
| 45 | Elemi res. | 639-99-6 487-11-6 | elemol elemicin |
| 46 | Galbanum res. | 489-86-1 51411-24-6 | guaiol dihydrofarnesol |
| 47 | Civet abs. | 542-46-1 | civetone |
| 48 | Castoreum abs. | 60125-06-6 1124-11-4 | isocastoramine tetramethylpyrazine |
| 49 | Citronella oil | 1117-61-9 106-24-1 106-23-0 | d-citronellol geraniol citronellal |
| 50 | Lemongrass oil | 5392-40-5 110-93-0 105-87-3 | citral methylheptenone geranyl acetate |
| 51 | Galbanum oil | 127-91-3 19883-29-5 | β-pinene undecatriene |
| 52 | Violet leaf abs. | 28069-72-9 557-48-2 | nonadienol nonadienal |
| 53 | Armoise oil | 546-80-5 464-49-3 | thujone camphor |
| 54 | Peppermint oil | 2216-51-5 89-80-5 89-48-5 | l-menthol menthone menthyl acetate |
| 55 | Spearmint oil | 6485-40-1 99-48-9 97-42-7 | l-carvone carveol carvyl acetate |
| 56 | Pine needle oil | 80-56-8 79-92-5 76-49-3 | α-pinene camphene bornyl acetate |
| 57 | Juniper berry oil | 80-56-8 127-91-3 | α-pinene β-pinene |
| 58 | Coriander oil | 78-70-6 464-49-3 507-70-0 | linalool camphor borneol |
| 59 | Celery seed oil | 5989-27-5 17066-67-0 6066-49-5 | d-limonene β-selinene 3-butylphthalide |
