# Supplementary material for: Identification of anti-SARS-CoV-2 agents based on flavor/fragrance compositions that inhibit the interaction between the virus receptor binding domain and human angiotensin converting enzyme 2
Source: PLoS One. 2022 Dec 19;17(12):e0279182. doi: 10.1371/journal.pone.0279182 (PMC9762593; doi:10.1371/journal.pone.0279182)
Supplement: S2 Table — The flavor/fragrance name and CAS number are shown. (DOCX) [file pone.0279182.s002.docx]

**S2 Table**

Synthetic flavor/fragrances.

| No. | Sample name | CAS number |
| --- | --- | --- |
| 60 | Aldehyde. C11(Lenic) (10-Undecenal) | 112-45-8 |
| 61 | Aldehyde. C12(Lauric)（dodecanal） | 112-54-9 |
| 62 | Aldehyde. C12 MNA（2-Methyl-undecanal） | 110-41-8 |
| 63 | Aldehyde. C10（n-Decanal） | 112-31-2 |
| 64 | Citronellol | 106-22-9 |
| 65 | Geraniol | 106-24-1 |
| 66 | Geranyl acetate | 105-87-3 |
| 67 | β-Damascone | 35044-68-9 |
| 68 | β-Phenylethyl alcohol | 60-12-8 |
| 69 | β-Phenylethyl acetate | 103-45-7 |
| 70 | Diphenyl oxide | 101-84-8 |
| 71 | L-Rose oxide | 16409-43-1 |
| 72 | Damascenone | 23696-85-7 |
| 73 | Linalool | 78-70-6 |
| 74 | Terpineol | 98-55-5 |
| 75 | Lily aldehyde | 80-54-6 |
| 76 | Lyral (registered brand) 4-(4-hydroxy-4-methylpentyl)cyclohex-3-ene-1-carbaldehyde） | 31906-04-4 |
| 77 | Benzyl acetate | 140-11-4 |
| 78 | α-Hexylcinnamaldehyde | 101-86-0 |
| 79 | Methyl dihydrojasmonate | 24851-98-7 |
| 80 | cis-Jasmone | 488-10-8 |
| 81 | Methyl β-naphthyl ketone | 93-08-3 |
| 82 | Aurantin (-)-6,7,7a,8-Tetrahydroquinazolino(3',2':1,6)pyrido(2,3-b)(1,4)benzodiazepine-9,16-dione | 522-16-7 |
| 83 | Methyl anthranilate | 134-20-3 |
| 84 | Linalyl acetate | 115-95-7 |
| 85 | Eugenol | 97-53-0 |
| 86 | Cinnamaldehyde | 104-55-2 |
| 87 | Methyl isoeugenol | 93-16-3 |
| 88 | Vertofix (registered brand) [3R-(3#,3a ,7 ,8a#)]-1-(2,3,4,7,8,8a-hexahydro-3,6,8,8-tetramethyl- 1H-3a,7-methanoazulen-5-yl)ethan-1-one） | 32388-55-9 |
| 89 | Santalex (registered brand) 4-(1,7,7-trimethyl-2-bicyclo[3.1.1]heptanyl)cyclohexan-1-ol） | 68877-29-2 |
| 90 | Bacdanol (registered brand) 2-Ethyl-4-(2,2,3-trimethylcyclopent-3-en-yl)-but-2-en-1-ol） | 28219-61-6 |
| 91 | Methyl atrarate | 4707-47-5 |
| 92 | α-Isomethyl ionone | 127-51-5 |
| 93 | α-Ionone | 127-41-3 |
| 94 | PTBCHA (p-tert-Butylcyclohexylacetate) | 32210-23-4 |
| 95 | Iso E super (registered brand) 1-(1,2,3,4,5,6,7,8-octahydro-2,3,8,8,-tetramethyl-2-naphthyl)ethan-1-one） | 54464-57-2 |
| 96 | Anethole | 104-46-1 |
| 97 | Anisaldehyde | 123-11-5 |
| 98 | cis-3-Hexenol | 928-96-1 |
| 99 | Triplal ([registered](https://eow.alc.co.jp/search?q=registered&ref=awlj) brand)：2,4-Dimethyl-3-cyclohexen-1-carbaldehyde | 27939-60-2 |
| 100 | Allyl amylglycolate | 67634-00-8 |
| 101 | Stemone （registered brand)：5-Methyl-3-heptanone Oxime | 22457-23-4 |
| 102 | 2-trans-6-cis-Nonadienol | 28069-72-9 |
| 103 | Styrallyl acetate | 93-92-5 |
| 104 | Phenylacetaldehyde DMA （Phenyl　acetaldehyde　dimethyl　acetal） | 101-48-4 |
| 105 | Helional （registered brand)：2-Methyl-3-(3,4-Methylenedioxyphenyl)Propanal | 1205-17-0 |
| 106 | cis-3-Hexenyl salicylate | 65405-77-8 |
| 107 | L-Menthol | 2216-51-5 |
| 108 | Camphor | 464-49-3 |
| 109 | Borneol | 507-70-0 |
| 110 | L-Carvone | 6485-40-1 |
| 111 | Musk ketone | 81-14-1 |
| 112 | Galaxolide （registered brand) 1,3,4,6,7,8-Hexahydro-4,6,6,7,8,8-hexamethylcyclopenta[g]-2-benzopyran） | 1222-05-5 |
| 113 | Pentalide （registered brand)：15-Pentadecanolide | 106-02-5 |
| 114 | Tonalid （registered brand)：6-Acetyl-1,1,2,4,4,7-hexamethyltetralin | 1506-02-1 |
| 115 | Indole | 120-72-9 |
| 116 | sec-Butyl quinoline | 67634-06-4 |
| 117 | Aldehyde. C14 （γ-Undecalactone） | 104-67-6 |
| 118 | Aldehyde. C16 （Ethyl methylphenylglycidate） | 77-83-8 |
| 119 | Aldehyde. C18 （γ-Nonalactone） | 104-61-0 |
| 120 | Raspberry ketone | 5471-51-2 |
| 121 | Maltol | 118-71-8 |
| 122 | Isoamyl acetate | 123-92-2 |
| 123 | Ethyl butyrate | 105-54-4 |
| 124 | Allyl caproate | 123-68-2 |
| 125 | Citral | 5392-40-5 |
| 126 | Citronellal | 106-23-0 |
| 127 | Citronellyl nitrile | 51566-62-2 |
| 128 | Nootkatone | 4674-50-4 |
| 129 | Dihydromyrcenol | 18479-58-8 |
| 130 | Vanillin | 121-33-5 |
| 131 | Ethyl vanillin | 121-32-4 |
| 132 | Coumarin | 91-64-5 |
| 133 | Heliotropin （1,3-Benzodioxole-5-carboxaldehyde） | 120-57-0 |
| 134 | Isoamyl salicylate | 87-20-7 |
| 135 | Cinnamyl alcohol | 104-54-1 |
| 136 | Methyl cinnamate | 103-26-4 |
| 137 | Ambroxan （registered brand) [3aR-(3aα,5aβ,9aα,9bβ)]-Dodecahydro-3a,6,6,9a-tetramethyl naphto[2,1-b]furan） | 6790-58-5 |
| 138 | Methyl salicylate | 119-36-8 |
| 139 | Methyl benzoate | 93-58-3 |
| 140 | Ethyl phenylacetate | 101-97-3 |
| 141 | Terpinyl acetate | 8007-35-0 |
| 142 | TCDA （Tricyclodecenylacetate） | 5413-60-5 |
| 143 | Isobornyl acetate | 125-12-2 |
| 144 | AMYL CINNAMIC ALDEHYDE | 122-40-7 |
| 145 | BENZYL SALICYLATE | 118-58-1 |
| 146 | CITRONELLYL ACETATE PURE | 150-84-5 |
| 147 | CYCLAMEN ALDEHYDE PURE | 103-95-7 |
| 148 | DAMASCONE DELTA (registered brand) 1-(2,6,6-Trimethyl-3-cyclohexen-1-yl)-2-buten-1-one） | 57378-68-4 |
| 149 | DMBC （Dimethylbenzylcarbinol） | 100-86-7 |
| 150 | DMBCA Dimethylbenzylcarbinyl acetate | 151-05-3 |
| 151 | DMPEC （Dimethylphenylethylcarbinol：2-METHYL-4-PHENYL-2-BUTANOL） | 10094-34-5 |
| 152 | DMBCB Dimethylbenzylcarbinyl butyrate | 72845-33-1 |
| 153 | ETHYL LINALOOL | 101-97-3 |
| 154 | ETHYL PHENYLACETATE | 17511-60-3 |
| 155 | TRICYCLODECENYL PROPIONATE | 2345-26-8 |
| 156 | GERANYL ISOBUTYRATE | 6259-76-3 |
| 157 | HEXYL SALICYLATE | 18871-14-2 |
| 158 | JASMAL | 106-25-2 |
| 159 | NEROL 900 | 60-12-8 |
| 160 | PHENYLETHYL ALCOHOL | 103-48-0 |
| 161 | PHENYL ETHYL ISO BUTYRATE | 103-48-0 |
| 162 | PHENYL ETHYL PHENYLACETATE | 102-20-5 |
| 163 | TERPINEYL ACETATE | 80-26-2 |
| 164 | TETRAHYDRO LINALOOL | 78-69-3 |
| 165 | ETHYLENE BRASSYLATE | 105-95-3 |
| 166 | GALAXOLIDE 50 BB | 1222-05-5 |
| 167 | CETONE V | 79-78-7 |
| 168 | Guaiyl acetate | 134-28-1 |
| 169 | METHYL CEDRYL ETHER | 19870-74-7 |
| 170 | VETIKOL ACETATE | 68083-58-9 |
| 171 | EBANOL （registered brand) 3-Methyl-5-(2,2,3-trimethyl-3-cyclopenten-1-yl)-4-penten-2-ol） | 67801-20-1 |
| 172 | ALLYL CYCLOHEXYL PROPIONATE | 2705-87-5 |
| 173 | ALLYL HEPTANOATE | 142-19-8 |
| 174 | APPLINAL | 6413-10-1 |
| 175 | BENZALDEHYDE | 100-52-7 |
| 176 | DELTA DECALACTONE | 705-86-2 |
| 177 | GAMMA DECALACTONE | 706-14-9 |
| 178 | ETHYL ISOBUTYRATE | 97-62-1 |
| 179 | ETHYL HEXANOATE | 123-66-0 |
| 180 | ETHYL 2-METHYLBUTYRATE | 7452-79-1 |
| 181 | ETHYL PROPIONATE | 105-37-3 |
| 182 | FRUITATE （registered brand) Ethyl tricyclo [5.2.1.02,6]decan-2-carboxylate） | 80657-64-3 |
| 183 | HEXYL ACETATE | 142-92-7 |
| 184 | OTBCHA | 88-41-5 |
| 185 | CIS-3-HEXENYL ACETATE | 3681-71-8 |
| 186 | UNDECAVERTOL （registered brand)：4-Methyl-3-decen-5-ol | 81782-77-6 |
| 187 | ALDEHYDE C-8 （registered brand)：Octanal | 124-13-0 |
| 188 | ALDEHYDE C-9 （registered brand)：n-Nonanal | 124-19-6 |
| 189 | FLOROPAL （registered brand)：2,4,6-trimethyl-4-phenyl-3-dioxane | 5182-36-5 |
| 190 | ESTRAGOLE | 140-67-0 |
| 191 | MENTHONE 85% UP | 89-80-5 |
| 192 | β-myrcene (myrcene) | 123-35-3 |
| 193 | γ-terpinene | 99-85-4 |
| 194 | (4R)-(+)-limonene (d-limonene) | 5989-27-5 |
| 195 | β-elemene | 33880-83-0 |
| 196 | α-pinene | 80-56-8 |
| 197 | β-pinene  (6,6-dimethyl-2-methylenebicyclo[3.1.1]heptane) | 127-91-3 |
| 198 | camphene  (2,2-dimethyl-3-methylenebicyclo[2.2.1]heptane) | 79-92-5 |
| 199 | 3-carene | 13466-78-9 |
| 200 | valencene | 4630-07-3 |
| 201 | β-guaiene (guaiene) | 88-84-6 |
| 202 | 1-isopropyl-4-methylbenzene (p-cymene) | 99-87-6 |
| 203 | farnesol | 4602-84-0 |
| 204 | nerolidol (3,7,11-trimethyl-1,6,10-dodecatrien-3-ol ) | 7212-44-4 |
| 205 | phytol | 150-86-7 |
| 206 | 4-isopropylbenzyl alcohol (cumic alcohol, p-cymen-7-ol) | 536-60-7 |
| 207 | 4-terpineol (terpinen-4-ol) | 562-74-3 |
| 208 | p-2,8-menthadien-1-ol | 22771-44-4 |
| 209 | carveol  (p-1(6),8-menthadien-2-ol) | 99-48-9 |
| 210 | elemol | 639-99-6 |
| 211 | verbenol | 473-67-6 |
| 212 | myrtenol | 515-00-4 |
| 213 | fenchol (fenchyl alcohol) | 1632-73-1 |
| 214 | β-caryophyllene alcohol | 472-97-9 |
| 215 | cedrol | 77-53-2 |
| 216 | (E,E)-2,4-hexadienal | 142-83-6 |
| 217 | (E,E)-2,4-decadienal | 25152-84-5 |
| 218 | β-Sinensal | 60066-88-8 |
| 219 | 3,4-dimethoxybenzaldehyde (veratraldehyde, methylvanillin) | 120-14-9 |
| 220 | 2-methoxycinnamaldehyde | 1504-74-1 |
| 221 | β-cyclocitral | 432-25-7 |
| 222 | safranal (2,6,6-trimethyl-1,3-cyclohexadienecarbaldehyde) | 116-26-7 |
| 223 | 3-(4-isopropylphenyl)-2-methylpropanal (cyclamen aldehyde) | 103-95-7 |
| 224 | myrtenal | 564-94-3 |
| 225 | geranylacetone | 3796-70-1 |
| 226 | isojasmone | 11050-62-7 |
| 227 | 5,6-epoxy-β-ionone | 23267-57-4 |
| 228 | 4-phenyl-3-buten-2-one | 122-57-6 |
| 229 | gingerone | 122-48-5 |
| 230 | pulegone | 89-82-7 |
| 231 | 1,10-dihydronootkatone | 20489-53-6 |
| 232 | 8,9-dehydrotheaspirone | 85248-56-2 |
| 233 | geranic acid | 459-80-3 |
| 234 | 4-hydroxy-3-methoxybenzoic acid (vanillic acid) | 121-34-6 |
| 235 | eugenyl acetate  (acetyl eugenol) | 93-28-7 |
| 236 | piperonyl acetate | 326-61-4 |
| 237 | isopentyl 2-hydroxybenzoate (isopentyl salicylate) | 87-20-7 |
| 238 | methyl N-methylanthranilate | 85-91-6 |
| 239 | ORIN LACTONE | 134359-15-2 |
| 240 | 5-(cis-3-Hexenyl)dihydro-5-methyl-2(3H)furanone | 70851-61-5 |
| 241 | Hexadec-7-en-1,16-lactone | 123-69-3 |
| 242 | dihydroactinidiolide | 15356-74-8 |
| 243 | 2-methoxy-3-methylpyrazine | 2847-30-5 |
| 244 | 2-isobutyl-3-methoxypyrazine | 24683-00-9 |
| 245 | 5-methylquinoxaline | 13708-12-8 |
| 246 | 4-methyl-5-thiazoleethanol acetic acid | 656-53-1 |
| 247 | 2,8-epithio-cis-p-menthane | 68398-18-5 |
| 248 | benzothiazole (1,3-benzothiazole) | 95-16-9 |
| 249 | 5-(2-hydroxyethyl)-4-methylthiazole | 137-00-8 |
| 250 | 1,1-Diethoxydecane | 34764-02-8 |
| 251 | 1,1-Diethoxy-3,7-dimethyl-2,6-octadiene | 7492-66-2 |
| 252 | 1,8-cineole (eucalyptol) | 470-82-6 |
| 253 | thymol (2-isopropyl-5-methylphenol) | 89-83-8 |
| 254 | carvacrol (5-isopropyl-2-methylphenol) | 499-75-2 |
| 255 | 1-allyl-4-methoxybenzene (estragole, methylchavicol) | 140-67-0 |
| 256 | isoeugenol (2-methoxy-4-(1-propenyl)-phenol) | 97-54-1 |
| 257 | methyleugenol (4-allyl-1,2-dimethoxybenzene) | 93-15-2 |
| 258 | methylisoeugenol (1,2-dimethoxy-4-(1-propenyl)benzene) | 93-16-3 |
| 259 | 3-(2-furyl)-2-propenal | 623-30-3 |
| 260 | limonene 1,2-oxide | 1195-92-2 |
| 261 | β-caryophyllene oxide | 1139-30-6 |
| 262 | theaspirane | 36431-72-8 |
| 263 | 3-(1-propylidene)-phthalide | 17369-59-4 |
| 264 | caryophyllene acetate | 57082-24-3 |
| 265 | Benzyl cinnamate | 103-41-3 |
| 266 | Cinnamyl cinnamate | 122-69-0 |
| 267 | Ethyl 3-phenylglycidate | 121-39-1 |
| 268 | Isoeugenyl acetate | 93-29-8 |
| 269 | Geranyl phenylacetate | 102-22-7 |
| 270 | Hydroxycitronellal diethyl acetal | 7779-94-4 |
| 271 | Methyl-α-ionone | 127-42-4 |
| 272 | Methyl 2-nonynoate | 111-80-8 |
| 273 | Octanal dimethyl acetal | 10022-28-3 |
| 274 | Propenylguaethol | 94-86-0 |
| 275 | Ethylene brassylate | 105-95-3 |
| 276 | Isoeugenyl benzyl ether | 120-11-6 |
| 277 | l-Menthyl lactate | 59259-38-0 |
| 278 | Vanillin isobutyrate | 20665-85-4 |
| 279 | Vanillyl butyl ether | 82654-98-6 |
| 280 | mono-Menthyl succinate | 77341-67-4 |
| 281 | Neohesperidin dihydrochalcone | 20702-77-6 |
| 282 | Ethyl vanillin propylene glycol acetal | 68527-76-4 |
| 283 | Vanillin propylene glycol acetal | 68527-74-2 |
| 284 | 2-(4-Methyl-5-thiazolyl)ethyl butanoate | 94159-31-6 |
| 285 | 2-(4-Methyl-5-thiazolyl)ethyl decanoate | 101426-31-7 |
| 286 | CINNAMALDEHYDE PROPYLENEGLYCOL ACETAL | 4353-01-9 |
| 287 | PIPERONAL PROPYLENEGLYCOL ACETAL | 61683-99-6 |
| 288 | Guaiyl acetate | 134-28-1 |
| 289 | Limonene diol | 38630-75-0 |
| 290 | α-Terpinyl ethyl ether | 27153-54-4 |
| 291 | Perilla ketone | 553-84-4 |
| 292 | Naringenin | 480-41-1 |
| 293 | Naringin Hydrate | 10236-47-2 |
| 294 | naringin Dihydrochalcone | 18916-17-1 |
| 295 | Hesperetin | 520-33-2 |
| 296 | Caffeic acid | 331-39-5 |
| 297 | 1-nonanol | 143-08-8 |
| 298 | (Z)-6-nonen-1-ol | 35854-86-5 |
| 299 | 3,6-nonadien-1-ol | 76649-25-7 |
| 300 | 1-decanol | 112-30-1 |
| 301 | (Z)-4-decen-1-ol | 57074-37-0 |
| 302 | 9-decen-1-ol | 13019-22-2 |
| 303 | perilla alcohol (p-1,8-menthadien-7-ol) | 536-59-4 |
| 304 | α-fenchol (α-fenchyl alcohol) | 14575-74-7 |
| 305 | (Z)-6-nonenal | 2277-19-2 |
| 306 | (E)-4-decenal | 65405-70-1 |
| 307 | hydroxycitronellal (7-hydroxy-3,7-dimethyloctanal) | 107-75-5 |
| 308 | 2-nonanone | 821-55-6 |
| 309 | 2-decanone | 693-54-9 |
| 310 | 3-methyl-1,2-cyclopentanedione (cyclotene) (CAS no [80-71-7]) | 765-70-8 |
| 311 | nonanoic acid (pelargonic acid) | 112-05-0 |
| 312 | octyl formate | 112-32-3 |
| 313 | ethyl 3-octenoate | 1117-65-3 |
| 314 | methyl 3-nonenoate | 13481-87-3 |
| 315 | 1,4-cineole | 470-67-7 |
| 316 | 2-pentylfuran | 3777-69-3 |
| 317 | 5-(hydroxymethyl)furfural | 67-47-0 |
| 318 | ocimene oxide | 69103-20-4 |
| 319 | carvone 1,2-oxide | 33204-74-9 |
| 320 | 2-phenoxyethanol | 122-99-6 |
| 321 | benzyl alcohol | 100-51-6 |
| 322 | 3-phenyl-1-propanol | 122-97-4 |
| 323 | p-1-menthen-9-ol | 18479-68-0 |
| 324 | 2-hydroxybenzaldehyde (salicylaldehyde) | 90-02-8 |
| 325 | 4-hydroxybenzaldehyde | 123-08-0 |
| 326 | linalool oxide pyranoid | 14049-11-7 |
| 327 | Benzaldehyde glyceryl acetal | 1319-88-6 |
| 328 | D-LIMONEN-10-OL | 38142-45-9 |
| 329 | Shogaol | 555-66-8 |
| 330 | dipropylene glycol | 25265-71-8 |
| 331 | isopropyl myristate | 110-27-0 |
